# 

**DOI:** 10.1055/s-0035-1570112

**Published:** 2016-01

**Authors:** Eduardo Camelo de Castro, Gercino Monteiro Filho, Waldemar Naves do Amaral

**Affiliations:** 1 Unidade de Saúde da Mulher do Curso de Medicina da Pontifícia Universidade Católica de Goiás; Diretor Associado da Humana Medicina Reprodutiva, Goiânia, GO, Brasil; 2 Faculdade de Medicina da Universidade Federal de Goiás, Goiânia, GO, Brasil; 3 Departamento de Ginecologia e Obstetrícia da Faculdade de Medicina da Universidade Federal de Goiás; Presidente da Sociedade Brasileira de Ultrassonografia, Goiânia, GO, Brasil

**Keywords:** ultrassonografia, folículo ovariano, ovário, infertilidade, ultrasonography, ovarian follicle, ovary, infertility

## Abstract

**Objetivos** Avaliar a variabilidade da contagem automática tridimensional dos folículos ovarianos que mediram 2 a 6 mm e 2 a 10 mm durante o ciclo menstrual. Verificar se este exame pode ser aplicado fora da fase folicular precoce do ciclo.

**Método** Prospectivo observacional. Foram incluídas todas as pacientes inférteis submetidas à monitorização da ovulação de 20 de abril de 2013 a 30 de outubro de 2014, com 18 a 35 anos; IMC de 18 a 25 kg/m ^2^ , eumenorréicas; sem história de cirurgia ovariana e sem alterações nas dosagens do TSH, prolactina, insulina e glicemia. Foram excluídas aquelas que apresentaram cistos ovarianos e as que faltaram algum dia da monitorização. A contagem ultrassonográfica dos folículos foi feita pelo modo 3D com SonoAVC na fase folicular precoce, folicular media, periovulatória e lútea do ciclo.

**Resultados** Quarenta e cinco mulheres foram incluídas. Houve diferença entre as médias das contagens dos folículos com 2 a 6 mm ( *p* = 0,001) e 2 a 10 mm ( *p* = 0,003) pelo teste de Friedman que avaliou conjuntamente as quatro fases do ciclo. Quando se aplicou o teste t-Student pareado, houve aumento significativo na contagem dos folículos de 2 a 6 mm quando se comparou a contagem desses folículos na fase folicular média e periovulatória com a contagem da fase lútea. Não houve diferença significante entre a contagem destes folículos pequenos nas fases folicular precoce, média e periovulatória.

**Conclusões** A variação da contagem automática tridimensional dos folículos de 2 a 6 mm, nas fases folicular precoce, folicular média e periovulatória, não mostrou significância estatística. Houve uma variação significativa da contagem automática 3D dos folículos ovarianos de 2 a 10 mm durante o ciclo. A variabilidade significativa da contagem dos folículos de 2 a 10 mm durante o ciclo não permite que este exame seja realizado fora da fase folicular precoce.

## Abstract (en)

**Objective** To evaluate the variability of three-dimensional automatic counts of ovarian follicles measuring 2–6 to 2–10 mm during the menstrual cycle and to determine if this test can be applied outside the early follicular phase of the menstrual cycle. Methods: in a prospective observational study, serial transvaginal ultrasound scans were performed from April 20, 2013, to October 30, 2014, on infertile patients. Inclusion criteria: age between 18 and 35 years, BMI 18–25 kg/m ^2^ , regular menstrual cycles, no history of ovarian surgery and no hormonal changes in TSH, prolactin, fasting insulin or glucose. We excluded patients with ovarian cysts or who did not complete one or more days of the serial transvaginal ultrasound scans. The follicle count was performed in 3D mode ultrasound with a SonoAVC system. Visits were scheduled for the early follicular, mid-follicular, periovulatory and luteal phases of the menstrual cycle.

**Results** Forty-five women were included. The Friedman test showed that the total number of follicles measuring 2–6 mm varied significantly ( *p* = 0.001) across the four periods of the menstrual cycle. The Paired Student *t* -test showed a significant increase in 2–6 mm follicle count from the mid-follicular and periovulatory phase to the luteal phase. We found no significant intra-cycle variation between the small follicles (2–6 mm) in the early follicular, mid-follicular and periovulatory phases. The Friedman test showed that the total number of follicles measuring 2–10 mm varied significantly ( *p* = 0.003) across the menstrual cycle.

**Conclusions** The variation of three-dimensional automatic counts of 2–6 mm follicles in the early follicular, mid-follicular and periovulatory phases was not statistically significant. The significant variability in the counts of follicles measuring 2–10 mm across the menstrual cycle does not permit this examination to be performed side the early follicular phase.

## Introdução

Folículos antrais são unidades histológicas do ovário que se formam após uma cavitação que ocorre na camada granulosa dos folículos pré-antrais. O número de folículos ovarianos que chegam ao estádio ovulatório é muito pequeno. Mais de 99% de todos os folículos sofrem um processo degenerativo apoptótico conhecido por atresia. Este processo pode ocorrer em qualquer estágio do desenvolvimento dos folículos e em qualquer fase do ciclo menstrual. Pela ultrassonografia é possível visualizar os folículos que têm antro e são maiores que 2 mm mas não é possível identificar quais estão em processo de atresia. Padronizou-se que a contagem ultrassonográfica dos folículos ovarianos deve incluir todas as imagens anecóicas, que medem 2 a 10 mm, e estão distribuídas no parênquima ovariano. 1

Foram publicados recentemente alguns trabalhos com a contagem ultrassonográfica dos folículos de 2 a 6 mm. Os autores justificaram que a somatória dos folículos ovarianos menores poderia melhorar o valor preditivo deste teste. 2 3 Por outro lado, uma padronização de 2010 já considerava que se devia contar os folículos de 2 a 10 mm de diâmetro para o laudo deste exame 1 ; mas mensuração manual do diâmetro de cada um dos folículos menores no modo 2D é muito trabalhosa. Além disso, já se conhecia uma variação da contagem dos folículos tanto quando realizada por um mesmo examinador em momentos distintos (intraobservador) como quando realizada por examinadores diferentes em uma mesma paciente (interobservador). Esta variação era maior ainda quando os examinadores tinham que avaliar o tamanho de cada folículo no momento em que estavam realizando a contagem. 1

Somente com o aperfeiçoamento recente das imagens ultrassonográficas e o desenvolvimento de um software dedicado foi possível fazer a contagem automática tridimensional dos folículos. A medida do tamanho e do volume dos folículos é mais precisa quando avaliada pela ultrassonografia tridimensional. Estudos recentes mostram que o duplo processamento das imagens proporciona alto grau de reprodutibilidade para a contagem ultrassonográfica dos folículos ovarianos, sendo superior às técnicas bidimensionais. 4

A maioria dos estudos sobre a confiabilidade e a validade dos vários testes da reserva ovariana é baseada em exames realizados durante a fase folicular precoce do ciclo menstrual. Esta janela de oportunidade relativamente pequena é restritiva tanto para os pacientes quanto para as clínicas que executam os testes. 5

Vários grupos têm sugerido uma boa estabilidade dos níveis do hormônio anti-Mülleriano ao longo do ciclo menstrual, 6 7 enquanto outros têm mostrado a presença de uma variação significativa deste hormônio no ciclo. 8 9 Ainda não há consenso sobre a variabilidade da contagem ultrassonográfica dos folículos ovarianos ao longo do ciclo. 10 Dessa forma, mais estudos são necessários para esclarecer se a contagem deve ser feita apenas na fase folicular precoce ou em qualquer fase do ciclo menstrual.

Os objetivos deste estudo foram avaliar a variabilidade da contagem automática tridimensional dos folículos ovarianos que mediram 2 a 6 mm e 2 a 10 mm durante o ciclo menstrual. Também teve o objetivo de verificar se este teste pode ser aplicado fora da fase folicular precoce do ciclo ovariano.

## Métodos

Foi feito um estudo prospectivo observacional no período de 20 de abril de 2013 a 30 de outubro de 2014, em uma clínica de Reprodução Assistida, a Humana Medicina Reprodutiva. O projeto foi aprovado pelo Comitê de Ética em Pesquisa do Hospital das Clínicas da Universidade Federal de Goiás.

Foram incluídas neste estudo, todas as pacientes que foram submetidas a monitorização da ovulação para avaliação do casal infértil, que estavam na faixa etária de 18 a 35 anos, tinham índice de massa corporal (IMC) entre 18 e 25 kg/m ^2^ , ciclos menstruais regulares com um intervalo de 26 a 32 dias, sem história de cirurgia ovariana e sem alterações hormonais sugestivas de doença endócrina como alteração da dosagem do TSH, prolactina, insulina de jejum e glicemia de jejum.

Foram excluídas deste estudo, as pacientes que apresentaram cistos ovarianos e as que não compareceram em um ou mais dias da monitorização da ovulação.

O primeiro dia do ciclo das pacientes foi contado a partir do primeiro dia de sangramento vivo da menstruação. A contagem ultrassonográfica dos folículos ovarianos foi realizada no início do ciclo menstrual (2° ao 5° dia), na fase folicular media (6° ao 10° dia), no período periovulatório (12° ao 16° dia) e na fase lútea (20° ao 26° dia). A avaliação da fase periovulatória teve início no 12° dia do ciclo ovariano. A partir do 12° dia foi feita monitorização diária até o instante que foi percebido um folículo dominante maior que 16 mm. Este dia foi considerado como momento periovulatório. A avaliação da fase lútea foi feita sete dias após este momento.

As ultrassonografias foram realizadas somente pelo pesquisador. Foi utilizado um equipamento Voluson® E6 (GE Healthcare) no modo tridimensional, sistema de alta resolução e ferramenta de automação (SonoAVC, GE Healthcare). As imagens foram captadas com transdutor transvaginal de 5 a 9 MHz.

A técnica ultrassonográfica empregada para a contagem e medição dos folículos ovarianos foi iniciada com o delineamento do ovário ainda no modo bidimensional. Esta imagem inicial foi selecionada com a digitação da tecla [3D] para gerar um volume tridimensional que englobou todo o ovário e excluiu as imagens extraovarianas. Uma vez que este conjunto de dados foi corretamente posicionado, o modo de varredura lenta do equipamento foi predefinido para percorrer um feixe com ângulo de 95°. Foi então digitado a tecla [Freeze] do ultrassom para a captação do volume ovariano. Neste momento, o sistema apresentou na tela do aparelho uma imagem que permitiu nova adequação da região tridimensional para inclusão de todo o ovário. Para a análise do volume selecionado, o botão [SonoAVC Follicles] foi digitado. Na nova tela de comandos, foi digitado [Right Ovary Start] para o ovário direito. Com isso foi exibida automaticamente, no canto superior esquerdo da tela do ultrassom, uma lista com a descrição dos volumes e dimensões correspondentes às imagens hipoecogênicas que pertenciam ao ovário e às regiões adjacentes. O contorno de cada folículo aparecia desenhado por cores diferentes. Todos os folículos detectados foram descritos e listados de acordo com o tamanho e a cor de cada um deles.

Após o processamento inicial das imagens, explicitado acima, fez-se um segundo processamento manual que consistiu na identificação e delimitação dos folículos que não foram contados e medidos automaticamente. Com o segundo processamento, também foi possível a exclusão de áreas hipoecogênicas adjacentes ao ovário que a imagem ultrassonográfica indicava que não eram pertencentes a este órgão. Essa verificação posterior foi feita para assegurar que todos os folículos haviam sido contados e medidos corretamente.

Para o ovário esquerdo foi realizada a mesma sequência. Porém, após a segunda seleção da região tridimensional de interesse, realizava-se a digitação de [Left Ovary Start] e em seguida iniciava-se o segundo processamento. O diâmetro médio de cada folículo dos dois ovários foi gravado e utilizado para análise posterior dos dados.

O Statistical Package for Social Sciences (versão 17.0; SPSS, EUA) foi utilizado para a análise estatística. O teste de Friedman foi aplicado para avaliar se havia uma variação significativa da contagem automática dos folículos ovarianos nas quatro fases do ciclo menstrual analisadas. O teste t-Student pareado foi utilizado para fazer a comparação entre duas fases do ciclo.

## Resultados

Foram submetidas a monitorização da ovulação no período do estudo 45 mulheres; o SonoAVC foi empregado em trezentos e sessenta ocasiões uma vez que dois ovários foram examinados em quatro fases do ciclo menstrual.

A média da idade das pacientes foi de 30,1 anos com desvio padrão de 3,9 e a média do IMC foi 22,6 com desvio padrão de 3,0 kg/m ^2^ .

A média, o desvio padrão e o intervalo de confiança das contagens dos folículos ovarianos, que mediram de 2 a 6 mm, nas quatro fases do ciclo menstrual, são mostrados na Tabela 1 . Houve diferença significante entre as médias das contagens após a aplicação do teste de Friedman ( *p* = 0,001) que avaliou conjuntamente a contagem dos folículos das quatro fases do ciclo ovariano ( Tabela 1 ).

**Tabela 1:** Contagem automática tridimensional dos folículos ovarianos de quarenta e cinco mulheres nas quatro fases do ciclo menstrual. Média, desvio padrão e intervalo de confiança do número de folículos que mediram de 2 a 6 mm

Fase do ciclo menstrual	Média	Desvio padrão	IC (95%)	
			Inferior	Superior
Folicular Precoce (2° ao 5° dia) ^A^	16,4	10,7	13,1	19,6
Folicular Média (6° ao 10° dia) ^B^	16,5	10,3	13,4	19,6
Periovulatória (12° - 16° dia) ^C^	16,3	10,1	13,3	19,4
Lútea (20° ao 26° dia) ^D; B; C^	18,6	11,5	15,1	22,0

Abreviações: IC, intervalo de confiança.

Teste: Friedman; *p* = 0,001. As letras iguais indicam que há diferença significativa entre as médias das duas fases do ciclo menstrual pelo teste t-Student pareado.

Uma segunda análise fez o pareamento das médias das contagens dos folículos ovarianos de 2 a 6 mm nas quatro fases do ciclo. Constatou-se que a média das contagens da fase lútea foi diferente da média das contagens do período periovulatório. Diferença semelhante foi verificada quando a média das contagens da fase lútea foi comparada com a da fase folicular média. O teste t-Student pareado mostrou que estas diferenças foram significantes. Por outro lado, quando foi feito o pareamento entre as outras demais fases do ciclo menstrual, não houve diferença estatística, ou seja, este teste não mostrou diferença entre as fases folicular precoce, média e periovulatória ( Tabela 1 ).

A média, o desvio padrão e o intervalo de confiança das contagens automáticas dos folículos, que mediram 2 a 10 mm, nas quatro fases do ciclo ovariano, podem ser visualizados na Tabela 2 . Nesta terceira análise dos dados foi possível perceber que houve diferença significante entre as médias das contagens dos folículos quando foram avaliadas conjuntamente as quatro fases do ciclo pelo teste de Friedman ( *p* = 0,003) ( Tabela 2 ).

**Tabela 2:** Contagem automática tridimensional dos folículos ovarianos de quarenta e cinco mulheres nas quatro fases do ciclo menstrual. Média, desvio padrão e intervalo de confiança do número de folículos que mediram de 2 a 10 mm

Fase do Ciclo Menstrual	Média	Desvio Padrão	IC (95%)	
			Inferior	Superior
Folicular Precoce (2° ao 5° dia) ^A^	20,2	11,5	16,7	23,6
Folicular Média (6° ao 10° dia) ^B;A^	22,0	11,9	18,4	25,6
Periovulatória (12° - 16° dia) ^C^	21,7	12,2	18,0	25,4
Lútea (20° ao 26° dia) ^D^	22,2	12,8	18,3	26,0

Abreviações: IC, intervalo de confiança.

Teste: Friedman; *p* = 0,003. As letras iguais indicam que há diferença significativa entre as médias das duas fases do ciclo menstrual pelo teste t-Student pareado.

Por último, foi realizado o pareamento das médias da contagem dos folículos de 2 a 10 mm nas quatro fases do ciclo ovulatório. Nesta análise foi possível perceber, pelo teste t-Student pareado, que a média das contagens da fase folicular precoce apresentou diferença estatisticamente significante com este mesmo dado calculado na fase folicular média ( Tabela 2 ).

## Discussão

No presente estudo, todas as contagens foliculares foram feitas por um examinador único e experiente para descartar a possibilidade da variação que pode acontecer nos laudos emitidos por observadores diferentes. Esta pesquisa não encontrou diferença na contagem automática tridimensional, dos folículos de 2 a 6 mm, realizada na fase folicular precoce, folicular média e periovulatória. Entretanto, houve aumento no número destes folículos quando os mesmos foram contados na fase lútea do ciclo. Houve variabilidade significativa da contagem dos folículos de 2 a 10 mm nas diferentes fases do ciclo.

Foram encontrados, até o momento, poucos estudos em que se analisou a variabilidade da contagem ultrassonográfica dos folículos ovarianos no ciclo menstrual. Desde o final da década passada, os autores já acreditavam que a maior variabilidade da contagem bidimensional dos folículos era decorrente de uma menor reprodutibilidade deste método. 1 Achava-se que a diferença intraobservador e interobservador da contagem ultrassonográfica dos folículos ovarianos poderia aumentar a diferença que havia nas medições realizadas nas diferentes fases do ciclo, comprometendo a versatilidade deste exame. 7

No final de 2007, especialistas em medicina reprodutiva se reuniram para sistematização dos conhecimentos sobre a contagem ultrassonográfica dos folículos ovarianos. 2 O objetivo da reunião foi padronizar a medição e a contagem dos folículos na prática clínica. Os autores desta padronização acreditavam que, durante a fase folicular precoce, os folículos ovarianos saudáveis mediam aproximadamente 4 a 6 mm de diâmetro. Acreditava-se que a contagem exclusiva desses folículos menores evitasse a inclusão de folículos atrésicos e a contagem superestimada de folículos ovarianos. Naquele momento, um estudo mostrava que a contagem de folículos pequenos (2 a 6 mm) tinha forte correlação com a contagem de folículos maiores (2 a 10 mm). 2 Ainda em 2009, propunha-se melhorar a reprodutibilidade da contagem ultrassonográfica dos folículos ovarianos deixando este exame menos dependente do examinador. 3

Em 2010, um trabalho pioneiro no qual se analisou a variação da contagem dos folículos ovarianos no ciclo menstrual, usando a ultrassonografia no modo 2D. Foi publicado um estudo prospectivo com quarenta e quatro mulheres. Estes autores foram os primeiros a estratificar a contagem dos folículos em tamanhos diferentes. O grupo concluiu que a diferença das contagens nas diversas fases do ciclo não eram grandes mesmo usando o modo manual 2D da ultrassonografia. Verificou-se que as flutuações das contagens, que ocorriam no ciclo, tinham pouca importância e que poderiam ser consideradas como achados fortuitos ou causadas por viés de mensuração. A contagem dos folículos de 2 a 5 mm mostrou maior variabilidade ao longo do ciclo ovulatório do que a contagem dos folículos de 2 a 10 mm e recomendaram a contagem destes folículos maiores para a avaliação da reserva ovariana. 7

Os resultados do presente estudo estão discordantes dos achados de van Disseldorp et al 7 já que mostraram menor variabilidade dos folículos ovarianos pequenos em comparação com os folículos totais (2 a 10 mm). Quanto à contagem dos folículos pequenos, somente a média da fase lútea foi diferente das outras três fases do ciclo menstrual. A diferença fundamental entre os dois trabalhos é que no presente estudo foi utilizado uma técnica ultrassonográfica tridimensional automática para a contagem dos folículos. Já foi demonstrado que este novo método melhora a reprodutibilidade da contagem dos folículos ovarianos. 11 Outros estudos já haviam mostrado que o modo 2D e outros métodos manuais podem superestimar o tamanho dos folículos. 3 11

Em uma padronização da contagem dos folículos ovarianos com ultrassonografia 2D foi recomendada a contagem dos folículos de 2 a 10 mm de diâmetro para evitar o processo moroso de medição de cada folículo por se acreditar que este seria um método mais prático para contagem dos folículos ovarianos na clínica. Preconizava também que a contagem dos folículos deveria ser feita na fase folicular precoce para minimizar a flutuação das medidas que poderiam ocorrer durante o ciclo menstrual. 1

Uma questão, ainda não respondida, é se a nova ultrassonografia tridimensional com software de automação melhorou a versatilidade da contagem dos folículos ovarianos após o conhecido aumento da reprodutibilidade que este exame proporcionou. Outro ponto importante é que a necessidade da contagem de folículos ovarianos menores aumenta muito a dificuldade do exame no modo 2D, principalmente nas mulheres que apresentam grande quantidade de folículos.

O único estudo, que avaliou os folículos ovarianos pela ultrassonografia, utilizando o SonoAVC, foi o de Deb et al. 5 Foram incluídas trinta e quatro mulheres na pesquisa. Os resultados sugeriram uma variação não significativa no número de folículos pequenos (2 a 6 mm) e uma excelente correlação entre as diferentes fases do ciclo menstrual. Os autores observaram ainda que a contagem dos folículos de 2 a 10 mm variou significativamente ao longo do ciclo. Os resultados mostraram que essa variação havia ocorrido predominantemente devido a uma alteração no número folículos com diâmetro maior que 6 mm e concluíram que a reserva ovariana avaliada pela contagem dos folículos pequenos mostrava menor variação no ciclo menstrual do que o volume ovariano, o FSH, o LH e o estradiol. 5

Os resultados do presente estudo, que também utilizou o SonoAVC, estão parcialmente de acordo com os resultados de Deb et al 5 já que não verificamos variação significativa no número de folículos ovarianos de 2 a 6 mm nas fases folicular precoce, folicular média e periovulatória. Entretanto houve um aumento no número destes folículos quando os mesmos foram contados na fase lútea do ciclo.

As diferenças dos resultados entre os trabalhos que utilizaram a ultrassonografia 2D e os estudos que estão utilizando a ultrassonografia 3D com automação está na melhor reprodutibilidade desta nova metodologia e a confiabilidade do novo método foi bem documentada1. 11 Assim observa-se que o aumento da reprodutibilidade da contagem dos folículos ovarianos, que ocorreu com esta técnica aumentou a versatilidade deste exame, ou seja, a contagem dos folículos de 2 a 6 mm que só podia ser avaliada na fase folicular precoce agora pode ser realizada também na fase folicular média e periovulatória. Por outro lado, como não há padronização para a contagem dos folículos de 2 a 6 mm, esse método ainda não deve ser usado para a classificação do perfil de resposta das pacientes nos ciclos de reprodução assistida.

Para se estabelecer as doses de gonadotrofinas exógenas usa-se contagem dos folículos de 2 a 10 mm que devem ser aplicadas nos ciclos de fertilização in vitro. 12 Sugere-se então que a individualização das doses seja feita na fase folicular precoce com base na contagem automática dos folículos de 2 a 10 mm.
